# A randomised clinical trial testing the safety of and metabolic responses to short-term duodenal infusion of recombinant RORDEP1 in healthy men

**DOI:** 10.1007/s00125-026-06751-0

**Published:** 2026-05-23

**Authors:** Joachim Gæde, Yong Fan, Liwei Lyu, Lærke Smidt Gasbjerg, Peter Rossing, Bolette Hartmann, Jens Juul Holst, Asger B. Lund, Filip K. Knop, Oluf Pedersen

**Affiliations:** 1 Center for Clinical Metabolic Research, Herlev and Gentofte University Hospital, University of Copenhagen, Hellerup, Denmark; 2 Novo Nordisk Foundation Center for Basic Metabolic Research, Faculty of Health and Medical Sciences, University of Copenhagen, Copenhagen, Denmark; 3 Department of Biomedical Sciences, Faculty of Health and Medical Sciences, University of Copenhagen, Copenhagen, Denmark; 4 Steno Diabetes Center Copenhagen, Herlev, Denmark; 5 Novo Nordisk A/S, Bagsværd, Denmark; 6 Department of Medicine, Faculty of Health and Medical Sciences, University of Copenhagen, Copenhagen, Denmark

**Keywords:** GIP, GLP-1, Glucose, Insulin, Metabolism, Microbiome, RORDEP1

## Abstract

**Aims/hypothesis:**

RUMTOR-derived peptides (RORDEPs) 1 and 2 are polypeptides synthesised by specific strains of the human gut commensal *Ruminococcus torques.* Preclinical studies have shown that RORDEPs lower blood glucose via an impact on plasma incretins and an improvement of hepatic insulin sensitivity. In a randomised, placebo-controlled, crossover trial, we here explore the safety and tolerability of, as well as any metabolic responses to, a duodenal infusion of recombinant RORDEP1 (r-RORDEP1) given to healthy men after oral intake of a liquid mixed meal.

**Methods:**

Seventeen healthy, normal-weight men between 18 and 35 years of age were randomised through block randomisation to receive either r-RORDEP1 or placebo as the initial intervention at Gentofte Hospital, Denmark. Exclusion criteria were use of any form of medication, use of antibiotics during the 3 months before intervention, lactose intolerance, smoking, alcohol or drug abuse, or the use of probiotics or creatine as dietary supplements during the study period. Blocks were created prior to trial initiation. Both participants and investigators were blinded to treatment. Following intake of a standardised liquid meal, r-RORDEP1 was given via a naso-duodenal tube as an initial bolus of 0.0108 mg/kg body weight followed by a continuous infusion of 0.25 µg kg^−1^ min^−1^ for 170 min. Primary outcomes were changes in plasma concentrations of incretins and peptide YY, while secondary endpoints were safety and tolerability, and changes in plasma insulin, C-peptide and glucose.

**Results:**

All 17 participants completed the trial. Duodenal infusion of r-RORDEP1 was well tolerated and without changes in biochemical measures of haematological, liver or renal functions. Compared with placebo, the bolus of r-RORDEP1 induced an early (at 15 or 30 min) rise in plasma glucagon-like peptide-1, insulin and C-peptide (*q*=0.001, *q*=0.001 and *q*=0.003, respectively) and a decline in plasma gastric inhibitory polypeptide and glucose (*q*=0.02 and *q*=0.006, respectively), while also increasing whole-body insulin sensitivity as measured with the Matsuda index of insulin sensitivity (*p*=0.049).

**Conclusions/interpretation:**

Short-term duodenal infusion of r-RORDEP1 is safe and well tolerated and elicits changes in plasma incretins, insulin and glucose, and a measure of whole-body insulin sensitivity, aligning with findings in rodents, supporting the hypothesis that RORDEPs hold a role in impacting host metabolism.

**Trial registration:**

ClinicalTrials.gov NCT06923839

**Funding:**

EFSD/Lilly European Diabetes Research Programme 2021, RUCILP F-19235-01-64 - NNF21SA0070428 grant and NNF23SA0084103 grant, the latter two from the Novo Nordisk Foundation.

**Graphical Abstract:**

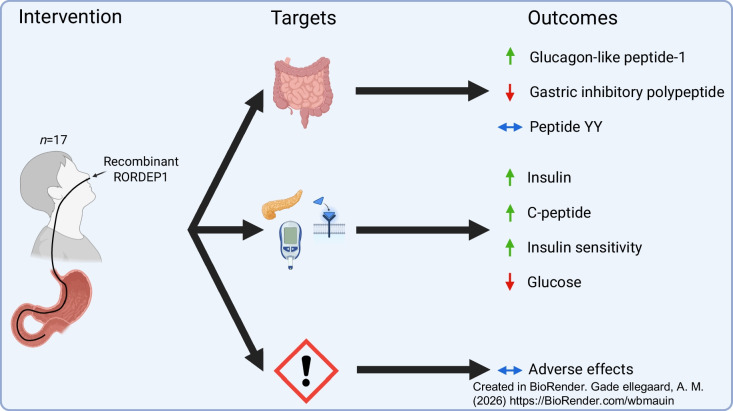

**Supplementary Information:**

The online version contains peer-reviewed but unedited supplementary material available at 10.1007/s00125-026-06751-0.


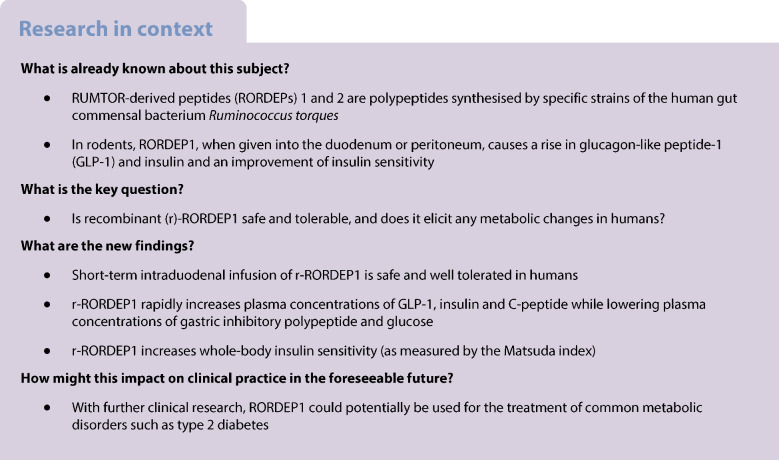


## Introduction

Recently, clinical interventions have shown that next-generation probiotics cultured from the human intestinal microbiome have the ability to affect various metabolic responses, such as glucose regulation and release of glucagon-like peptide-1 (GLP-1) [1–3]. Though the molecular mechanisms responsible for these metabolic changes are difficult to decipher due to the complex interconnectedness of the individual probiotics with the vast and complex gut microbiome, it has, based on studies in humans and rodents, been suggested that some of the peptides produced by next-generation probiotics may impact host metabolism. For example, Amuc_1100 and P9 proteins synthesised by *Akkermansia muciniphila* [4] and RUMTOR-derived peptides (RORDEPs) 1 and 2 synthesised by specific strains of *Ruminococcus torques* have been shown to exert regulatory effects on metabolism [5].

RORDEP1 and 2 are cleaved from the precursor protein RUMTOR_00181 and have lengths of 87 and 88 amino acids, respectively, with 73% amino acid identity. The biological activities of the two polypeptides are similar [5]. RORDEPs are thought to mediate their biological effects via the enteric nervous system but both circulate in human blood with plasma concentrations around 170–210 pmol/l [5]. In studies of rats wherein recombinant RORDEP1 (r-RORDEP1) was given intraperitoneally there was an increase in plasma GLP-1, peptide YY (PYY) and insulin and a decline in plasma gastric inhibitory polypeptide (GIP) and glucose [5]. When r-RORDEP1 was given into the duodenum of rats, the blood glucose declined due to a 40% reduction in insulin-mediated inhibition of hepatic gluconeogenesis [5].

Clinically, RORDEP1 has been indirectly investigated through a 6 h randomised clinical trial in which the live RORDEP1-producing *R. torques* ATCC 27756 strain was infused into the duodenum of healthy, overweight adults (Gæde J, Fan Y et al, unpublished). In that study, the *R. torques* strain was shown to increase plasma concentrations of GLP-1 and PYY, while decreasing plasma concentrations of GIP, mirroring previous findings in rodents treated with r-RORDEP1 [5].

To further explore the safety and tolerability and the potential short-term metabolic effects of r-RORDEP1 in humans, we conducted a randomised, double-blind, placebo-controlled crossover trial in normal-weight and normoglycaemic young men. In this trial, r-RORDEP1 or placebo, after intake of a standardised liquid mixed meal, was given as a duodenal bolus followed by a continuous duodenal infusion for 170 min. Due to the first-in-human nature of our clinical study, we conducted a toxicity study in mice as a precaution to confirm the safety of the intervention with r-RORDEP1 before initiation of the clinical trial.

## Methods

### Study outline

**Fig. 1:**
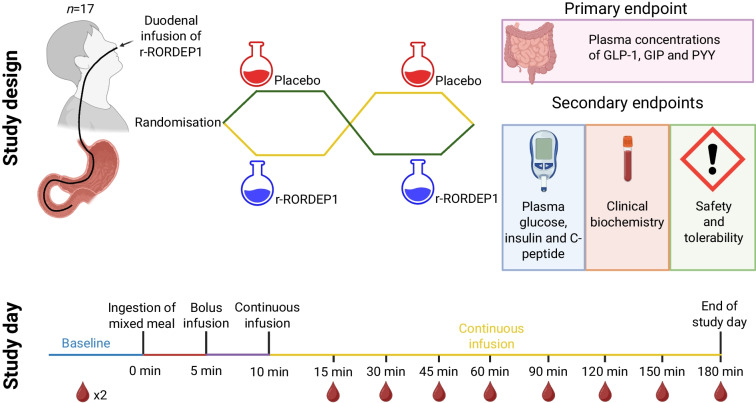
Trial overview, including design, study day and endpoints. The liquid mixed meal (Nutridrink compact, Nutricia) consisted of 250 ml, 2510 kJ, 23.3 g of fat, 74.3 g of carbohydrates and 24.0 g of protein. The duodenal infusion dosing regimen was as follows: a bolus of 0.0108 mg r-RORDEP1/kg body weight was infused over 5 min followed by a continuous infusion of 0.25 µg kg^−1^ min^−1^ for the rest of the study period (170 min). Blood sampling times are shown on the timeline. Clinical biochemistry measurements were done at baseline and at the end of the MMT (180 min). Created in BioRender. Gade ellegaard, A. M. (2026) https://BioRender.com/k5bhkj1

The study was conducted at the Center for Clinical Metabolic Research, Department of Medicine, Herlev-Gentofte University Hospital, Copenhagen between July and September 2025. It was designed as a randomised, placebo-controlled, double-blind crossover trial. An overview of the study is provided in Fig. 1.

Participants were randomised to receive either r-RORDEP1 or placebo as the first intervention or vice versa. All participants received both interventions.

Randomisation was done through a randomisation sheet using block randomisation and participants were randomised on their first study day. Both investigators and participants were blinded to treatment, although investigators had access to a blinded randomisation sheet to facilitate administration of the intervention. Each of the two study days were at least 5 days apart to allow for a wash-out period, minimising the risk for carry-over effects.

The study protocol was approved by the Ethics Committee of Capital Region of Denmark (registration number H-22011935). The maximal duodenal bolus dose of ‘Generally Recognised As Safe’ (GRAS)-quality r-RORDEP1 endorsed by the Ethics Committee of Capital Region of Denmark was 0.0108 mg/kg body weight. The approved maximal continuous duodenal infusion rate over 170 min was 0.25 µg kg^−1^ min^−1^. The trial was registered at ClinicalTrials.gov (registration no. NCT06923839).

The study was conducted in accordance with the Helsinki Declaration and written informed consent was obtained from all participants prior to screening and trial enrolment.

### Study participants

Seventeen self-reported Danish men were included in the study. Inclusion criteria were age 18–35 years, normal BMI (18.5 kg/m^2^ to <25 kg/m^2^) and absence of acute or chronic disease. Exclusion criteria were use of any form of medication, use of antibiotics during the 3 months before intervention, lactose intolerance, smoking, alcohol or drug abuse, or the use of probiotics or creatine as dietary supplements during the study period.

During the inclusion period (April–August 2025), 33 individuals gave informed consent and underwent a screening process involving anthropometric and biochemical profiling. Of these individuals, 24 were included in the study. However, seven individuals withdrew their informed consent due to the study conflicting with summer vacation, leaving 17 individuals for participation in the study. No participants dropped out during the trial (electronic supplementary material [ESM] Fig. 1).

### GRAS-compliant production of non-His-tagged r-RORDEP1

For detailed information on the production of GRAS-compliant non-His-tagged r-RORDEP1, see ESM Methods.

### Primary and secondary trial endpoints

Our previous clinical study investigating the effects of the RORDEP1-producing gut bacterium *R. torques* strain ATCC 27756 in humans showed significant increases in the AUC of plasma GLP-1 and PYY and a significant decrease in the AUC of plasma GIP (Gæde J, Fan Y et al, unpublished). We therefore chose changes in plasma concentrations of GLP-1, GIP and PYY at any point during the 3 h time course of the mixed meal test (MMT) as the primary composite endpoint.

Secondary endpoints included changes in plasma insulin, C-peptide and glucose concentrations, as well as a surrogate measure of whole-body insulin sensitivity, as these variables have in previous rodent studies been affected by an intervention with r-RORDEP1 [5]. Further, tolerability and safety endpoints were self-reported adverse effects (both during the study day and the following 4 days after intervention) as well as clinical biochemical markers (blood leukocytes, eosinophils, neutrophils, platelets, haemoglobin, plasma concentrations of alanine aminotransferase [ALT], aspartate aminotransferase [AST], alkaline phosphatase, albumin, creatinine, sodium, potassium, calcium, phosphate, creatine kinase and C-reactive protein [CRP]).

### Intervention and blood sampling

Study participants arrived in the morning at the facility in an overnight-fasted state (minimum 10 h) after having ingested a carbohydrate-rich meal the evening before. Study participants were further asked not to consume alcohol or do vigorous physical activity during the 48 h leading up to each of the two study days.

Participants were weighed when they arrived at the facility, and fasting blood samples were subsequently taken. A naso-duodenal tube was then installed using the CORTRAK-2 Enteral Access System (Avanos), a placement technique successfully used in our previous study (Gæde J, Fan Y et al, unpublished). After the placement of the naso-duodenal tube, participants ingested a liquid mixed meal orally (Nutridrink Compact, Nutricia; 250 ml, 2510 kJ (600 kcal), containing 23.3 g of fat, 74.3 g of carbohydrates and 24.0 g of protein) during a maximum of 5 min. After the 5 min, a bolus of r-RORDEP1 or placebo (identical to the active solution except for the absence of r-RORDEP1) was given. The bolus consisted of 0.0108 mg r-RORDEP1 per kg body weight (in a volume of 100 ml isotonic saline (154 mmol/l NaCl, Fresenius KABI) containing 5% (wt/vol.) human albumin (CLS Behring)). This bolus was infused into the duodenum over 5 min. During the following 170 min, the continuous infusion rate of r-RORDEP1 was 0.25 µg kg^−1^ min^−1^, resulting in a total duration of the observed MMT of 180 min (Fig. 1).

The vials containing the two interventional compounds were visually identical and had identical viscosity and smell, allowing for investigators to be blinded while administering the intervention.

Blood was sampled twice at baseline, and then every 15 min during the first hour of the MMT, and every 30 min during the remaining experimental period (180 min in total).

### Analysis of plasma hormones

Venous blood was collected at the prespecified time points in chilled EDTA tubes containing 10 µl valine pyrrolidide (1 mmol/l) per ml of blood. The tubes were kept on ice until centrifuging no later than 30 min after the blood was collected. Plasma was then collected and stored at −20°C (for analysis of GLP-1, GIP and PYY) or −80°C (for analysis of insulin, C-peptide and glucose). Insulin and C-peptide were measured using Siemens Atellica IM analyzer. Total GLP-1, GIP and PYY were analysed as previously described [6–8].

### Calculating a Matsuda index from plasma glucose and insulin excursions following a standardised liquid mixed meal

The Matsuda index was developed using data from an OGTT, specifically plasma glucose and insulin levels at fasting (time 0), 30 min, 60 min, 90 min and 120 min. This index estimates whole-body insulin sensitivity by combining baseline (predominantly hepatic) and post-load (predominantly muscle/fat) responses, correlating well with the gold-standard insulin clamp method [9]. In the present study with ingestion of a liquid mixed meal instead of oral glucose, we calculated a Matsuda index using the algorithm provided from the official website (https://mmatsuda.diabetes-smc.jp/english.html, accessed 21 November 2025).

### Analysis of standard biochemical variables

Haematological variables (i.e. blood total leukocytes, eosinophils, lymphocytes, neutrophils, platelets and haemoglobin) were analysed using ADVIA 2120i 3 M, while plasma ALT, AST, alkaline phosphatase, albumin, creatinine, sodium, potassium, calcium, phosphate, creatine kinase, HbA_1c_ and plasma glucose were analysed using Siemens Atellica CH 930.

### Analysis of adverse effects

Adverse effects were defined as any biochemical abnormalities in blood samples or any experienced side effects mentioned in a diary. Biochemical markers were assessed after the MMT and at a follow-up visit on average 7 days after the MMT. This assessment time was chosen for practical reasons to enable the first follow-up visit to overlap with the second study day, thus reducing the number of visits to the research facility for the participants. In practice though, some participants were unable to actualise the follow-up exactly 7 days after due to various personal reasons and the number of days between follow-ups ranged between 5 and 16.

During the first 4 days after each intervention day, participants were asked to fill out a diary in the evening before bedtime to explore whether any adverse effects occurred following the intervention. Participants were asked to note in the diary whether they experienced onset/worsening of the following symptoms: nausea; vomiting; flatulence; bloating; borborygmi; reflux; diarrhoea; constipation; abdominal pains or cramps; headache; dizziness; sleep disturbances; fatigue; pruritus; and skin rash. Participants were likewise asked to mark on a visual analogue scale (VAS) their general wellbeing during the day in question.

Due to the short half-life of r-RORDEP1 in simulated intestinal fluid of about 3 h [5], any side effects would most likely occur during the first 2 days but we chose to follow the participants with a diary for 4 days as a precaution.

### Statistics

The dose of r-RORDEP1 used in the clinical part of the study was found by converting doses from previous rodent studies (see ESM Methods: Converting doses of r-RORDEP1 from rats to humans). Using the statistical software R(https://www.r-project.org/, version 4.5.0, function power.t.test), we calculated, with a power of 0.9, a required sample size of 13 to detect a change in plasma GIP concentration of 14%, which we considered to be of physiological relevance. We aimed to have 15 participants finish the study to ensure adequate statistical power. Seventeen men completed the trial (ESM Fig. 1), enabling us to detect a similar change in plasma GLP-1 and a change in plasma PYY of 18%.

The statistical tools GraphPad Prism and R were used for statistical analysis. Baseline values used in the statistical analyses for GLP-1, GIP, PYY, insulin, C-peptide and glucose were calculated as the mean of the two baseline samples taken. For comparisons of AUCs, a paired *t* test was used. For comparisons between time points during the MMT, we used a two-way ANOVA and report the resulting *q* values defined as *p* values corrected for multiple testing using Šidák correction. For analysis of data on adverse effects from the diaries, a general linear mixed model (function glmer, package ‘lme4’) with Poisson distribution was used. Further, paired *t* tests were used to compare the clinical biomarkers both at the end of the MMT and at the follow-up visit.

All 17 participants were included in all analyses of primary and secondary endpoints except for the following analyses:

- two participants were excluded from analysis of GIP and GLP-1 due to missing values on all time points due to a technical error
- two participants were excluded from analysis of creatine kinase at the end of the MMT due to having elevated values at baseline
- one participant was excluded from analysis of ALT and AST at follow-up due to having consumed alcohol the day before blood sampling
- two participants were excluded from analysis of creatine kinase at follow-up due to having been too physically active leading up to blood sampling.

### Mouse study

Even though RORDEP1 is continuously synthesised by the intestinal microbiome of healthy individuals and circulates in human plasma [5], we decided to test for any evidence of r-RORDEP1 toxicity in short-term studies of mice; experiments were carried out at GenScript Biotech Biotech Co. (Nanjing, China).

Specific pathogen-free (SPF) male C57BL/6J mice (*Mus musculus*; international strain nomenclature: C57BL/6J) were obtained from Beijing Vital River Laboratory Animal Technology Co. (Beijing, China; Laboratory Animal Production License no. SCXK [Jing] 2021-0011 and SCXK [Jing] 2021-0006). Mice were 8–9 weeks old at study initiation, with a body weight range of 18–35 g. Animals were acclimatised prior to experimentation and housed under standard SPF conditions.

Fourteen male mice were initially screened, and 12 mice meeting predefined inclusion criteria were enrolled in the study. Inclusion criteria required body weights within 80–120% of the group mean and absence of any clinical abnormalities. Animals outside this range or showing signs of ill health were excluded prior to randomisation; no animals required exclusion after study initiation. Mice were randomly assigned to two groups (*n*=6 per group).

Six mice received a single intraperitoneal injection of r-RORDEP1 at 2 mg/kg body weight, and six mice received PBS as a control. The dose of r-RORDEP1 was chosen based on previous studies in similar mice where r-RORDEP1 was injected intraperitoneally for 7 days at a dose of 1 mg/kg per day, eliciting a major increase in the expression of genes involved in thermogenesis and browning of inguinal white adipose tissue [5]. In the toxicity study, mice were monitored daily for any signs that could be related to a change of behaviour. After 7 days, the mice were euthanised. Blood was examined for differences between groups in haematology, liver and kidney biomarkers. Necropsy was performed with organ weighing, gross pathology and histopathology of liver, gallbladder, spleen, heart, lung, kidneys and main bronchus.

## Results

### Study participants

Baseline characteristics for the 17 healthy men who completed the study are shown in ESM Table 1. All participants were healthy and had a BMI within the normal range (18.5 kg/m^2^ to <25 kg/m^2^). Participants did not use any medication during the study.

### Toxicity experiments in mice

r-RORDEP1 was well tolerated in the mouse study, no death or moribundity (i.e. the animal being close to death) occurred in either group and no abnormalities or between-group differences in food intake, body weight or any of the endpoints were observed (data not shown). Based on the outcomes of the toxicity experiments, we considered GRAS-quality r-RORDEP1 with a sequence of amino acids identical to native RORDEP1 as a safe polypeptide to be delivered into duodenum of healthy young individuals.

### Measures of tolerability and safety in the human trial

r-RORDEP1 was well tolerated. The study participants did not report any significantly higher occurrences of any of the side effects noted in the diaries during the 4 days of follow-up after an intervention (ESM Table 2), nor were there any adverse effects that resulted in dropout or hospitalisation of participants. At the end of the MMT, we observed a slight increase in blood platelets and at the follow-up visit minor increases in haemoglobin and plasma calcium levels after the intervention with r-RORDEP1 (ESM Tables 3, 4). We consider the observed minor changes to bear no physiological relevance.

### Metabolic effects of r-RORDEP1

**Fig. 2:**
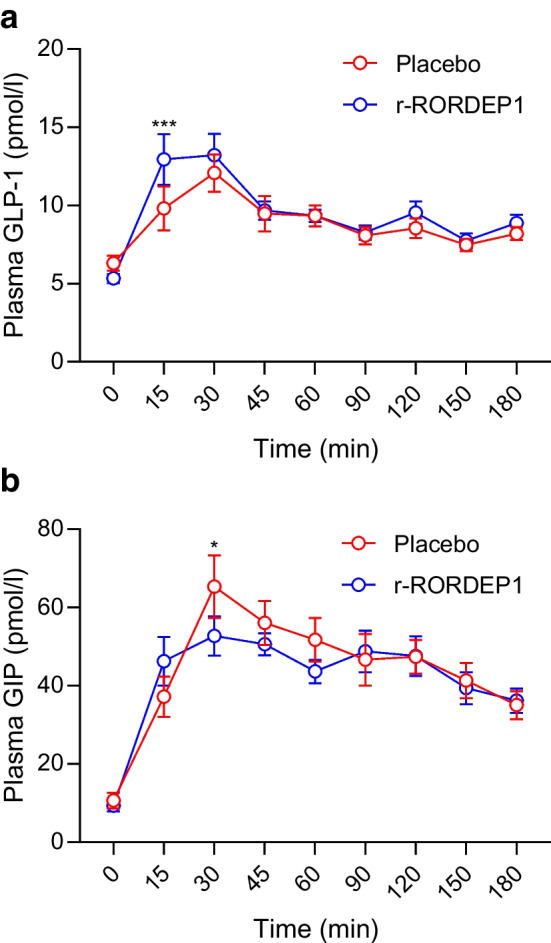
Plasma concentrations of GLP-1 and GIP during the MMT. (**a**) Plasma GLP-1 concentrations during the MMT. At 15 min, plasma GLP-1 was 3.1 pmol/l higher after intervention with r-RORDEP1 (95% CI 1.0, 5.3; *q*=0.001). (**b**) Plasma GIP concentrations during the MMT. At 30 min, plasma GIP was 12.6 pmol/l lower after intervention with r-RORDEP1 (95% CI 1.0, 24.2; *q*=0.02). Comparisons between placebo and RORDEP1 at the different time points were done using two-way ANOVA. The reported *q* values are defined as *p* values adjusted for multiple testing using Šidák correction. Circles and bars represent mean and SEM. *q<0.05, ***q<0.001

**Fig. 3:**
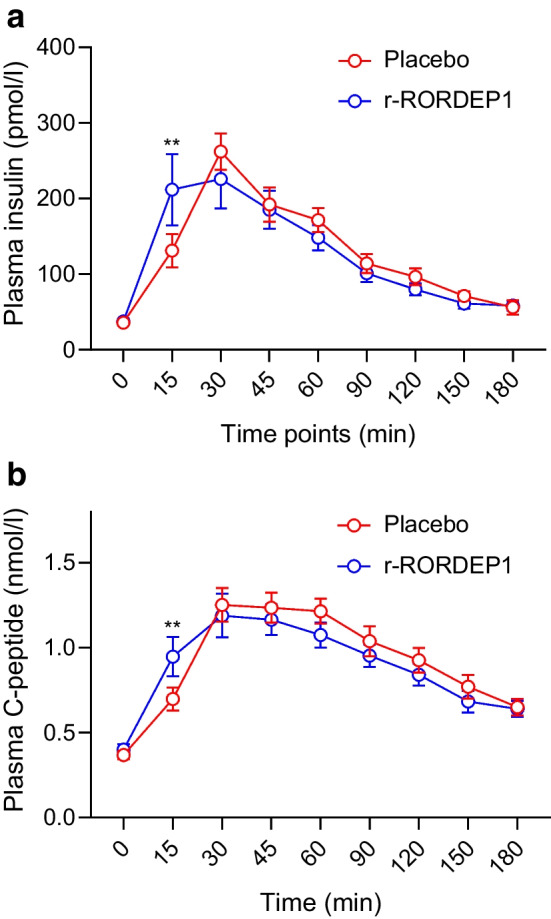
Plasma concentrations of insulin and C-peptide during the MMT. (**a**) Plasma insulin concentrations during the MMT. At 15 min, plasma insulin was 80.6 pmol/l higher after intervention with r-RORDEP1 (95% CI 23.6, 137.6; *q*=0.001). (**b**) Plasma C-peptide concentrations during the MMT. At 15 min, plasma C-peptide was 0.25 nmol/l higher after intervention with r-RORDEP1 (95% CI 0.06, 0.44; *q*=0.003). Comparisons between placebo and RORDEP1 at the different time points were done using two-way ANOVA. The reported *q* values are defined as *p* values adjusted for multiple testing using Šidák correction. Circles and bars represent mean and SEM. **q<0.01

**Fig. 4:**
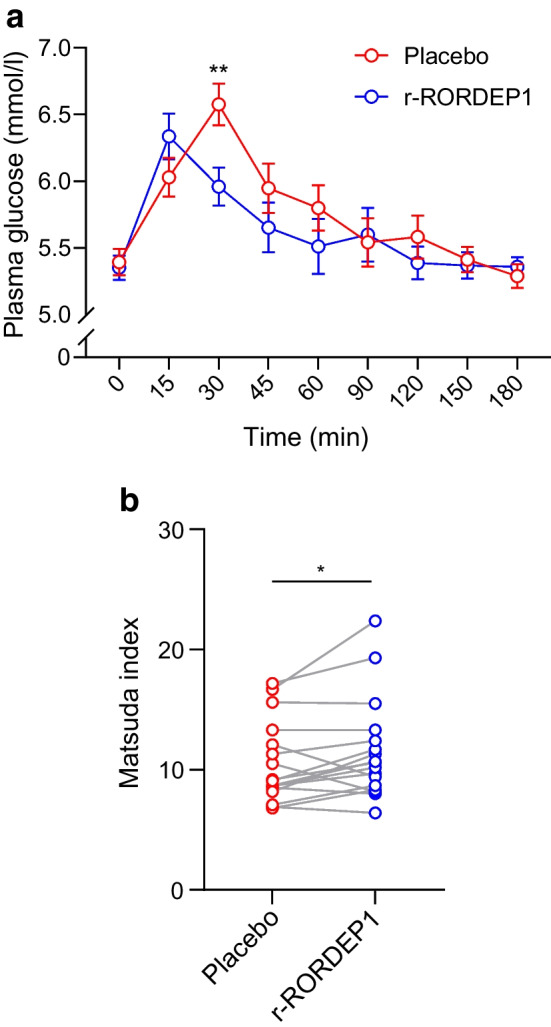
Plasma concentrations of glucose during the MMT and Matsuda index of insulin sensitivity. (**a**) Plasma glucose concentrations during the MMT. At 30 min, plasma glucose was 0.6 mmol/l lower after intervention with r-RORDEP1 (95% CI 0.1, 1.1; *q*=0.0055). Comparisons between placebo and RORDEP1 at the different time points were done using two-way ANOVA. The reported *q* values are defined as *p* values adjusted for multiple testing using Šidák correction. (**b**) Matsuda index during treatment with placebo and RORDEP1. The Matsuda index was 1.0 arbitrary units higher after intervention with r-RORDEP1 (95% CI 0.005, 2.1; *p*=0.0491). Circles and bars represent mean and SEM in (**a**) and circles represent individual values in (**b**). *p<0.05, **q<0.01

Compared with placebo, at the early time points (at 15 or 30 min) of the MMT the bolus of r-RORDEP1 induced a mean rise in plasma GLP-1 of 3.1 pmol/l (95% CI 1.0, 5.3; *q*=0.001), in plasma insulin of 80.6 pmol/l (95% CI, 23.6, 137.6; *q*=0.001) and in plasma C-peptide of 0.25 nmol/l (95% CI 0.06, 0.44; *q*=0.003) and a mean decline in plasma GIP of 12.6 pmol/l (95% CI 1.0, 24.2; *q*=0.02) and in plasma glucose of 0.6 mmol/l (95% CI 0.1, 1.1; *q*=0.006) (Figs 2, 3 and 4). No differences between placebo and r-RORDEP1 were found in the AUCs of the MMT (ESM Table 5). Plasma PYY concentrations were comparable between the two treatment groups (ESM Fig. 2).

Interestingly, insulin sensitivity, assessed with the Matsuda index, was significantly increased after the liquid mixed meal and duodenal infusion of r-RORDEP1 compared with placebo (*p*=0.049, Fig. 4).

## Discussion

In this randomised, placebo-controlled, double-blind, crossover feasibility trial in 17 young healthy men, we investigated the tolerability and safety of the r-RORDEP1 polypeptide and its potential metabolic effects after a liquid mixed meal. The duodenal infusion of an r-RORDEP1 bolus followed up by 170 min of a continuous infusion of r-RORDEP1 was safe and without self-reported or biochemical adverse effects when comparing biochemical markers of organ functions. Compared with placebo, we found an increase in plasma GLP-1 10 min after initiation of the r-RORDEP1 bolus infusion (15 min after the ingestion of the liquid meal). In parallel with the increase in plasma GLP-1, an increase in both plasma insulin and C-peptide occurred. The observation might be a result of the rise in plasma GLP-1, which is known to increase plasma insulin levels [10]. However, it could also be due to an intrinsic ability of r-RORDEP1 to increase insulin release directly, possibly via the small pool of insulin-filled granules in the pancreatic beta cells [11]; this release is known to suppress hepatic glucose production [12]. Indeed, plasma glucose at 30 min following the liquid meal was decreased. In addition, we found an increase in the Matsuda index, suggesting an increase of whole-body insulin sensitivity following duodenal infusion of r-RORDEP1. In parallel with the decline in plasma glucose, we found a decline in plasma GIP; a similar reduction was found 30 min after i.p. injection of r-RORDEP1 in rodents [5]. Any clinical or therapeutic relevance of these results is hard to assess given the study population of healthy, normal-weight men. Yet, a 0.6 mmol/l reduction in plasma glucose might be of clinical importance if the effect was sustained through the entire postprandial period. A reduction in plasma glucose of roughly the same size is reported by Hjerpsted et al during the first h after a meal test following a 12-week treatment with semaglutide [13]. However, due to the restricted study population and the isolated and transient effects on the reported endpoints, caution should be taken when interpreting the interesting but limited results.

The changes in intestinal and pancreatic hormone release at the initial time points and the improvement in insulin sensitivity induced by a bolus of r-RORDEP1 in healthy men align with preclinical findings [5]. However, in rats, a 12-fold higher r-RORDEP1 bolus dose was needed to elicit an increase in plasma concentrations of GLP-1 and insulin and a decrease in GIP 45 min after an i.p. injection of r-RORDEP1 (see ESM Methods: Converting doses of r-RORDEP1 from rats to humans; see also [5]). Therefore, our current findings may suggest that the sensitivity to r-RORDEP1 is considerably higher in humans than in rodents. Whether this species-related difference in RORDEP1 sensitivity is related to the fact that the intestinal microbiome of rats does not contain strains of *R. torques* that synthesise RORDEPs remains unknown.

The continuous very low infusion rate of r-RORDEP1 (yet the maximal infusion rate endorsed by the Ethics Committee of Capital Region of Denmark for this first-in-human trial) did not trigger any metabolic responses. Likely, the lack of measurable bioactivity of the low dosing is explained by the rapid degradation of the polypeptide by intestinal proteases [5].

In simulated intestinal fluid, r-RORDEP1 is estimated to have a half-life of around 3 h [5]. However, based on the observations in the present real-life human study, this estimate seems inconsistent with the short duration of the metabolic effects of the r-RORDEP1 bolus. Since RORDEP1 appears to elicit its effects from the gut lumen [5], one way to circumvent this challenge might be to administer r-RORDEP1 in slow-release, acid-resistant capsules, which would also enable longer-term interventions. Another way would be to chemically modify the r-RORDEP1 protein to have a longer half-life.

Our trial has limitations. First, our trial is short-term (only 3 h). Second, as discussed, the amount of r-RORDEP1 given in the present first-in-human trial and endorsed by medical ethical authorities is modest compared with the r-RORDEP1 doses that are needed to elicit metabolic effects in rodents. Therefore, future long-term randomised clinical trials of the safety, tolerability and metabolic efficacy of r-RORDEP1 should be carried out with escalating dosing of r-RORDEP1 formulated in acid-resistant and slow-release capsules. Third, we were unable to standardise the follow-up to exactly 7 days for all participants, thus introducing some uncertainty into the interpretation of the clinical biomarkers at follow-up. However, most participants managed to have their follow-up visit between 6 and 8 days after the study day and so we would still argue that the study fulfils its purpose as a feasibility study. Finally, since this is a first-in-human study, the participants were limited to healthy young men, which limits the generalisability of the study. To establish the potential therapeutic value of r-RORDEP1, further randomised clinical trials need to be undertaken with a broader population (e.g. individuals with type 2 diabetes or impaired glucose tolerance).

### Conclusions and perspectives

In this short-term safety and feasibility trial in healthy men, we show that duodenal infusion of r-RORDEP1 is safe and well tolerated and improves measures of insulin release and whole-body insulin sensitivity in accordance with recent observations in rats [5]. Long-term human interventions with escalating dosing of enteric coated r-RORDEP1 capsules are warranted to explore long-term tolerability and safety as well as effects on metabolism.

## Supplementary Information

Below is the link to the electronic supplementary material.

ESM (PDF 317 KB)

## Data Availability

Data analysed in the current study are not publicly available due to data protection regulations but are available from the corresponding author upon reasonable request starting from the date of publication and for a minimum of 5 years. Data will be shared with qualified researchers addressing research questions meeting legal and ethical requirements. Data will be shared in anonymised form as Excel sheets.

## Abbreviations

ALT: Alanine aminotransferase
AST: Aspartate aminotransferase
GIP: Gastric inhibitory polypeptide
GLP-1: Glucagon-like peptide-1
MMT: Mixed meal test
PYY: Peptide YY
RORDEP: RUMTOR-derived peptide
r-RORDEP1: Recombinant RORDEP1
