# Supplementary material for: A randomised clinical trial testing the safety of and metabolic responses to short-term duodenal infusion of recombinant RORDEP1 in healthy men
Source: Diabetologia. 2026 May 23;69(8):2338–46. doi: 10.1007/s00125-026-06751-0 (PMC13310194; doi:10.1007/s00125-026-06751-0)
Supplement: Supplementary file 1 — ESM (PDF 317 KB) [file 125_2026_6751_MOESM1_ESM.pdf]

Electronic supplementary methods

GRAS-compliant production of non-His-tagged r-RORDEP1

Non-His-tagged r-RORDEP1 was manufactured under ‘Generally Recognized as Safe’ (GRAS)–compliant conditions by GenScript Biotech Co. (Nanjing, China). Production was performed using a non-pathogenic, well-characterised *Escherichia coli* BL21 Star (DE3) expression strain harbouring the recombinant plasmid encoding RORDEP1 under the control of a T7 RNA polymerase–dependent inducible promoter system. Recombinant protein expression was initiated by the addition of isopropyl β-D-1-thiogalactopyranoside (IPTG). The host strain and inducible expression system were selected based on their extensive history of safe use in recombinant protein production and suitability for GRAS-aligned manufacturing. All raw materials were non-animal-derived and of high purity, and all procedures were conducted using sterilised equipment within an ISO 9001-certified quality management system, in accordance with U.S. Food and Drug Administration GRAS guidance.

The overall production process is divided into upstream (fermentation) and downstream (purification) processes. Upstream processing comprised sequential seed culture expansion, controlled fermentation, and biomass harvesting. A single vial from the primary cell bank, consisting of a 6×His-tagged RORDEP1–expressing *E. coli* BL21 Star (DE3) strain, was thawed at room temperature and inoculated into Lysogeny Broth (LB) medium for primary seed expansion. Cultivation was performed at 37 °C with constant agitation at 4.5-7.0 g, and cell growth was monitored by measuring optical density at 600 nm (OD600) until mid-log phase was reached. The primary seed culture was then transferred into antibiotic-free terrific broth (TB) medium for secondary seed expansion under controlled temperature and agitation. Large-scale fermentation was conducted in a sterilised stainless-steel fermenter containing antibiotic-free TB medium supplemented with feeding solution, antifoam 204, and 10% ammonium hydroxide for pH control. Fermentation temperature was maintained within the range of 30–37 °C, and dissolved oxygen

levels were regulated by adjusting airflow, agitation speed, and tank pressure. When the culture reached the target cell density corresponding to an OD600 of approximately 20–30, r-RORDEP1 expression was induced by addition of IPTG to a final concentration of 0.5 mmol/L. Controlled nutrient feeding was initiated following induction, and OD600 was measured both immediately prior to induction and at the end of fermentation. Cells were harvested by centrifugation, and the wet biomass was weighed to ensure batch consistency.

Downstream processing began with bacterial lysis and clarification. Harvested cell pellets were resuspended in resuspension buffer (Buffer A) and lysed by sonication using a protocol of 600 W power with 3 seconds on and 6 seconds off cycles for a total sonication time of 20 min, performed in an ice bath to minimise heat-induced protein degradation. The lysis procedure was repeated twice to ensure efficient cell disruption. Lysates were clarified by centrifugation, and the resulting supernatant was further filtered through a polyethersulfone (PES) capsule filter. The clarified lysate was then subjected to affinity chromatography using resin pre-equilibrated in Buffer C (150 mmol/L NaCl). After sample loading, the column was washed with Buffer B to remove non-specifically bound proteins. Gradient elution was performed sequentially using Buffer D and Buffer E, and eluted fractions were analysed by bicinchoninic acid (BCA) assay and Sodium dodecyl sulfate-polyacrylamide gel electrophoresis (SDS–PAGE) to confirm protein concentration and identity. Fractions containing r-RORDEP1 were pooled for subsequent processing.

To obtain the final non-His-tagged product, histidine affinity tag removal was achieved by enzymatic cleavage using Tobacco Etch Virus (TEV) protease. Protein concentration of the pooled fractions was determined by BCA assay, and TEV protease was added at a ratio of 0.05 mL protease per mg of recombinant protein. The cleavage reaction was carried out at 30 °C for 16 h under controlled conditions. Following cleavage, the reaction mixture was dialysed against Buffer C (150 mmol/L NaCl) and passed through a second affinity chromatography step to remove the cleaved tag and protease, with the tag-free r-RORDEP1 collected in the flow-through. Final buffer exchange was performed using a 3.5 kDa molecular-weight-cutoff dialysis membrane against the

final formulation buffer, with a minimum dilution factor of 1:1000 and a total dialysis duration of at least 18 hours, including two buffer changes. Protein concentration and purity were verified by BCA assay and SDS–PAGE prior to sterile filtration.

The purified r-RORDEP1 solution was sterilised by filtration through a 0.22 μm membrane under aseptic conditions and aliquoted into sterile 15 mL vials, with each vial filled with 7 mL of protein solution. Lyophilisation was performed using a validated freeze-drying protocol following thorough cleaning and sanitisation of the freeze-dryer with 75% ethanol. The resulting lyophilised r-RORDEP1 powder was labelled, packaged, and stored at −70 °C ± 10 °C until use. Prior to trial starting day, the lyophilised protein was reconstituted in phosphate-buffered saline (PBS, pH 7.4). The entire manufacturing process adhered to Food and Drug Administration (FDA)-defined GRAS requirements, with continuous quality oversight ensuring product safety, purity, and reproducibility.

Final quality control testing demonstrated that the non-His-tagged r-RORDEP1 met all predefined release specifications, with each parameter evaluated using validated analytical methods and corresponding results as follows. Protein purity was assessed by reducing SDS-PAGE, showing a purity of 98.8%, and independently confirmed by reversed-phase high-performance liquid chromatography (RP-HPLC) with a purity of 97.94%. Monomer content was determined by size exclusion (SEC)-HPLC, yielding 99.34% monomer. Protein identity and molecular weight were verified by liquid chromatography-mass spectrometry (LC-MS), which was consistent with the theoretical molecular weight (9,244.39 Da). Protein quantity was measured by BCA assay, with a yield of 5.8 mg per bottle. Endotoxin levels of the final r-RORDEP1 product were quantified using a kinetic chromogenic limulus amebocyte lysate (LAL) assay and were <0.217 endotoxin units (EU)/mg. Host cell protein contamination was measured by ELISA and found to be <8.696 ng/mg. Bioburden testing by membrane filtration met all criteria, including total aerobic microbial count $<10^3$ cfu/mL, total yeast and mould count $<10^2$ cfu/mL, and absence of *E. coli* in 1 mL of final product. Residual His-tag and residual small ubiquitin-like modifier (SUMO) protease were assessed by anti-His Western blotting and were not detected. Residual heavy metals were

analysed by inductively coupled plasma optical emission spectroscopy (ICP-OES), with cadmium <0.01 mg/kg, arsenic <0.05 mg/kg, mercury <0.01 mg/kg, nickel 0.0606 mg/kg, and lead <0.10 mg/kg. Residual kanamycin and IPTG were quantified by liquid chromatography-tandem mass spectrometry (LC-MS/MS) and were <0.20 µg/g and <1.00 µg/g, respectively.

Converting doses of r-RORDEP1 from rats to humans

To translate the intraduodenal infusion dose of r-RORDEP1 used in rats to a corresponding human dose, we applied body surface area (BSA) normalisation. This approach is widely used for interspecies dose conversion and accounts for differences in metabolic rate and physiological scaling associated with body size (PMID: 27057123). Based on the standard BSA translation algorithm, the Km factor is 6 for rats and 37 for humans, yielding a rat-to-human conversion factor of approximately 6.2.

To calculate the Human Equivalent Dose (HED) we divided the optimal dose in rats of 200 pmol/kg/min with the conversion factor to get 32.26 pmol/kg/min. Based on a molecular weight of 9,244 Da for RORDEP1, this corresponds to a total administered dose of 0.0533 mg/kg body weight over a 175-minute infusion period.

The full infusion protocol consists of a bolus over 5 minutes delivering 20% of the total amount of r-RORDEP1 followed by a steady infusion of the remaining 80% of r-RORDEP1 over 170 minutes. This corresponds in humans to a bolus infusion rate of 2.16 µg/kg/min for 5 minutes, followed by a continuous infusion rate of 0.25 µg/kg/min for 170 minutes. Under this protocol, the total administered dose is 0.0533 mg/kg of r-RORDEP1.

In the present human study, the bolus dose was set to 0.0108 mg/kg. Using BSA-based back-conversion, this dose corresponds approximately to 67 µg/kg in rats. Importantly, this is substantially lower than the 800 µg/kg dose previously administered via intraperitoneal injection in a separate rat study (PMID: 40745048), representing roughly one-twelfth of that exposure.

ESM Table 1

Baseline characteristics	
Age (years)	27 (23; 28)
BMI (kg/m^2)	22.6 (21.3; 23.6)
Alcohol (units/week)	3 (2; 5)
HbA1c (mmol/mol)	31 (29; 33)
HbA1c (%)	5.0 (4.8; 5.2)
b-leukocytes (10^9/l)	5.1 (4.7; 5.6)
b-eosinophils (10^9/l)	0.13 (0.07; 0.14)
b-lymphocytes (10^9/l)	1.66 (1.41; 1.85)
b-neutrophils (10^9/l)	2.87 (2.45; 3.31)
b-platelets (10^9/l)	218 (190; 250)
b-haemoglobin (g/l)	147 (143; 156)
p-ALT (µkat/l)	0.4 (0.3; 0.5)
p-AST (µkat/l)	0.4 (0.3; 0.4)
p-alkaline phosphatase (µkat/l)	1.4 (1.2; 1.5)
p-albumin (g/L)	45 (43; 48)
p-creatinine (µmol/L)	69 (67; 81)
p-sodium (mmol/L)	142 (141; 142)
p-potassium (mmol/L)	4.1 (3.9; 4.3)
p-calcium (mmol/L)	2.53 (2.48; 2.62)
p-phosphate (mmol/L)	1.11 (1.00; 1.15)
p-creatine kinase (µkat/l)	3.2 (2.3; 5.3)
p-CRP (mg/L)	All <4

Legend to ESM Table 1: Baseline characteristics.

Values are median (interquartile range).

ALT, alanine aminotransferase; AST, aspartate aminotransferase; b, blood; p, plasma.

ESM Table 2

Adverse effects during the four days of follow-up				
	Placebo	r-RORDEP1	Difference (95% CI)	p-value
Nausea (counts)	0	2	NA	NA
Vomiting (counts)	0	0	NA	NA
Flatulence (counts)	7	10	43% (-45%; 293%)	0.469
Bloating (counts)	5	7	40% (-55%; 373%)	0.566
Stomach cramps (counts)	0	2	NA	NA
Rumbling in the stomach (counts)	5	13	160% (-2%; 710%)	0.069
Reflux (counts)	0	1	NA	NA
Stomach pains (counts)	2	4	NA	NA
Diarrhoea (counts)	3	1	NA	NA
Constipation (counts)	1	3	NA	NA
Headache (counts)	4	6	50% (-57%; 487%)	0.530
Dizziness (counts)	2	6	200% (-30%; 1947%)	0.178
Insomnia (counts)	1	2	NA	NA
Tiredness (counts)	8	10	25% (-51%; 227%)	0.638
Itchiness (counts)	0	0	NA	NA
Rash (counts)	0	0	NA	NA
VAS	7.2	6.2	-0.3 (-1.0; 0.5)	0.469

Legend to ESM Table 2: experienced side effects during the four-day follow-up period.

Data shown are the number of days where any of the listed side effects are experienced/worsened during the follow-up period of four days after the intervention. Data were analysed using a generalised mixed model with a Poisson distribution.

Missing values are due to insufficient occurrences for the specific side effect in question.

VAS, visual analogue scale.

ESM Table 3

Blood biomarkers at end of the MMT				
	Placebo	r-RORDEP1	Difference (95% CI)	p-value
b-leukocytes (10^9/l)	6.1 (5.4; 7.4)	5.4 (5.1; 6.1)	-0.5 (-1.3; 0.2)	0.163
b-eosinophils* (10^9/l)	0.10 (0.06; 0.18)	0.10 (0.06; 0.17)	NA	0.745
b-lymphocytes (10^9/l)	1.63 (1.37; 1.83)	1.62 (1.35; 1.86)	0.01 (-0.07; 0.10)	0.720
b-neutrophils (10^9/l)	3.76 (2.92; 5.25)	3.06 (2.63; 3.94)	-0.50 (-1.28; 0.27)	0.187
b-platelets* (10^9/l)	205 (176; 227)	217 (190; 232)	NA	**0.019**
b-haemoglobin* (g/l)	139 (133; 145)	139 (132; 143)	NA	0.769
p-ALT (µkat/l)	0.3 (0.3; 0.4)	0.3 (0.3; 0.5)	0.03 (-0.01; 0.07)	0.131
p-AST* (µkat/l)	0.3 (0.3; 0.4)	0.4 (0.3; 0.5)	NA	0.310
p-alkaline phosphatase (µkat/l)	1.1 (0.9; 1.3)	1.1 (0.9; 1.3)	0.02 (-0.01; 0.05)	0.220
p-albumin (g/L)	40 (39; 40)	39 (38; 40)	-0.2 (-0.9; 0.6)	0.617
p-creatinine (µmol/L)	67 (61; 71)	66 (62; 72)	-0.2 (-2.9; 2.5)	0.855
p-sodium (mmol/L)	140 (139; 142)	141 (140; 142)	0.2 (-0.5; 0.9)	0.482
p-potassium (mmol/L)	3.8 (3.7; 4.0)	3.8 (3.7; 4.0)	0.04 (-0.06; 0.14)	0.378
p-calcium (mmol/L)	2.36 (2.31; 2.40)	2.33 (2.29; 2.37)	-0.01 (-0.04; 0.01)	0.378
p-phosphate (mmol/L)	1.11 (1.00; 1.20)	1.08 (1.01; 1.18)	0.01 (-0.05; 0.08)	0.651
p-creatine kinase* (µkat/l)	2.0 (1.4; 2.7)	2.5 (1.8; 2.9)	NA	0.366
p-CRP† (mg/L)	All <4	All <4	NA	NA

Legend to ESM Table 3: biochemical safety markers at end of the MMT

Data are shown as median (interquartile range) and as effect size (95% CI). Significant p-values are marked in bold. Data are analysed with paired t-tests when normally distributed and with Wilcoxon test when not normally distributed.

MMT, mixed meal test; CI, confidence interval; ALT, alanine aminotransferase; AST, aspartate aminotransferase; b, blood; p plasma.

* Wilcoxon test used

† All results were undetectable and therefore noted as <4. Statistical test was not performed.

ESM Table 4

Blood biomarkers at follow-up				
	Placebo	r-RORDEP1	Difference (95% CI)	p-value
b-leukocytes (10^9/l)	5.2 (4.7; 6.2)	5.2 (4.4; 5.6)	-0.2 (-1.0; 0.6)	0.637
b-eosinophils (10^9/l)	0.11 (0.08; 0.18)	0.11 (0.07; 0.18)	-0.02 (-0.05; 0.01)	0.216
b-lymphocytes (10^9/l)	1.83 (1.56; 1.98)	1.79 (1.66; 2.03)	0.02 (-0.14; 0.19)	0.769
b-neutrophils (10^9/l)	2.65 (2.33; 3.48)	2.70 (2.21; 3.10)	-0.22 (-0.92; 0.49)	0.524
b-platelets (10^9/l)	234 (192; 246)	222 (201; 262)	0.4 (-11; 12)	0.940
b-haemoglobin (g/l)	142 (132; 150)	143 (139; 150)	3.1 (0.2; 6.0)	**0.037**
p-ALT (µkat/l)	0.3 (0.3; 0.4)	0.4 (0.3; 0.4)	0.01 (-0.04; 0.06)	0.592
p-AST* (µkat/l)	0.4 (0.3; 0.4)	0.4 (0.3; 0.5)	NA	0.993
p-alkaline phosphatase (µkat/l)	1.2 (0.9; 1.4)	1.2 (0.7; 1.5)	0.00 (-0.09; 0.09)	>0.999
p-albumin (g/mol)	43 (41; 45)	44 (44; 45)	1.2 (-0.06; 2.5)	0.060
p-creatinine* (µmol/L)	73 (68; 82)	79 (68; 84)	NA	0.140
p-sodium (mmol/L)	140 (139; 142)	140 (140; 142)	0.1 (-1; 1)	0.835
p-potassium (mmol/L)	3.8 (3.6; 3.9)	3.8 (3.6; 4.0)	0 (-0.2; 0.2)	>0.999
p-calcium (mmol/L)	2.41 (2.34; 2.46)	2.43 (2.40; 2.49)	0.04 (0.0003; 0.07)	**0.048**
p-phosphate (mmol/L)	1.16 (0.96; 1.23)	1.08 (0.91; 1.19)	-0.02 (-0.11; 0.06)	0.547
p-creatine kinase* (µkat/l)	3.3 (2.3; 3.9)	2.9 (1.9; 4.3)	NA	0.890
CRP† (mg/L)	All <4	All <4	NA	NA

Legend to ESM Table 4: biochemical safety markers at follow-up.

Data are shown as median (interquartile range) and as effect size (95% CI). Significant p-values are marked in bold. Data are analysed with paired t-tests when normally distributed and with Wilcoxon test when not normally distributed.

CI, confidence interval; ALT, alanine aminotransferase; AST, aspartate aminotransferase; b, blood; p, plasma.

* Wilcoxon test used

† All results were undetectable and therefore noted as <4. Statistical test was not performed.

ESM Table 5

Area under the curve				
	Placebo	r-RORDEP1	Difference	p-value
p-GLP-1 ((pmol/l)×min)	1572 (1398; 1747)	1686 (1515; 1857)	114 (-4; 232)	0.058
p-GIP ((pmol/l)×min)	8199 (6428; 9970)	7902 (6593; 9219)	-297 (-1611; 1016)	0.635
p-PYY ((pmol/l)×min)	2124 (1734; 2514)	2326 (1930; 2721)	202 (-7; 411)	0.057
p-glucose ((mmol/l)×min)	1015 (973; 1057)	1004 (972; 1037)	-11 (-62; 41)	0.668
p-insulin ((pmol/l)×min)	22135 (19172; 25098)	21083 (17956; 24209)	-1052 (-3167; 1063)	0.307
p-C-peptide ((nmol/l)×min)	170.4 (150.3; 190.4)	161.6 (143.3; 180.0)	-8.7 (-21.1; 3.7)	0.155

Legend to ESM Table 5: Area under the curve for primary and secondary endpoints.

Data are shown as mean (95% confidence interval of the mean) and as effect size (95% confidence interval). Comparisons were done using paired t-tests.

GLP-1, Glucagon-Like Peptide-1; GIP, Gastric Inhibitory Polypeptide; PYY, Peptide YY.

ESM Fig. 1

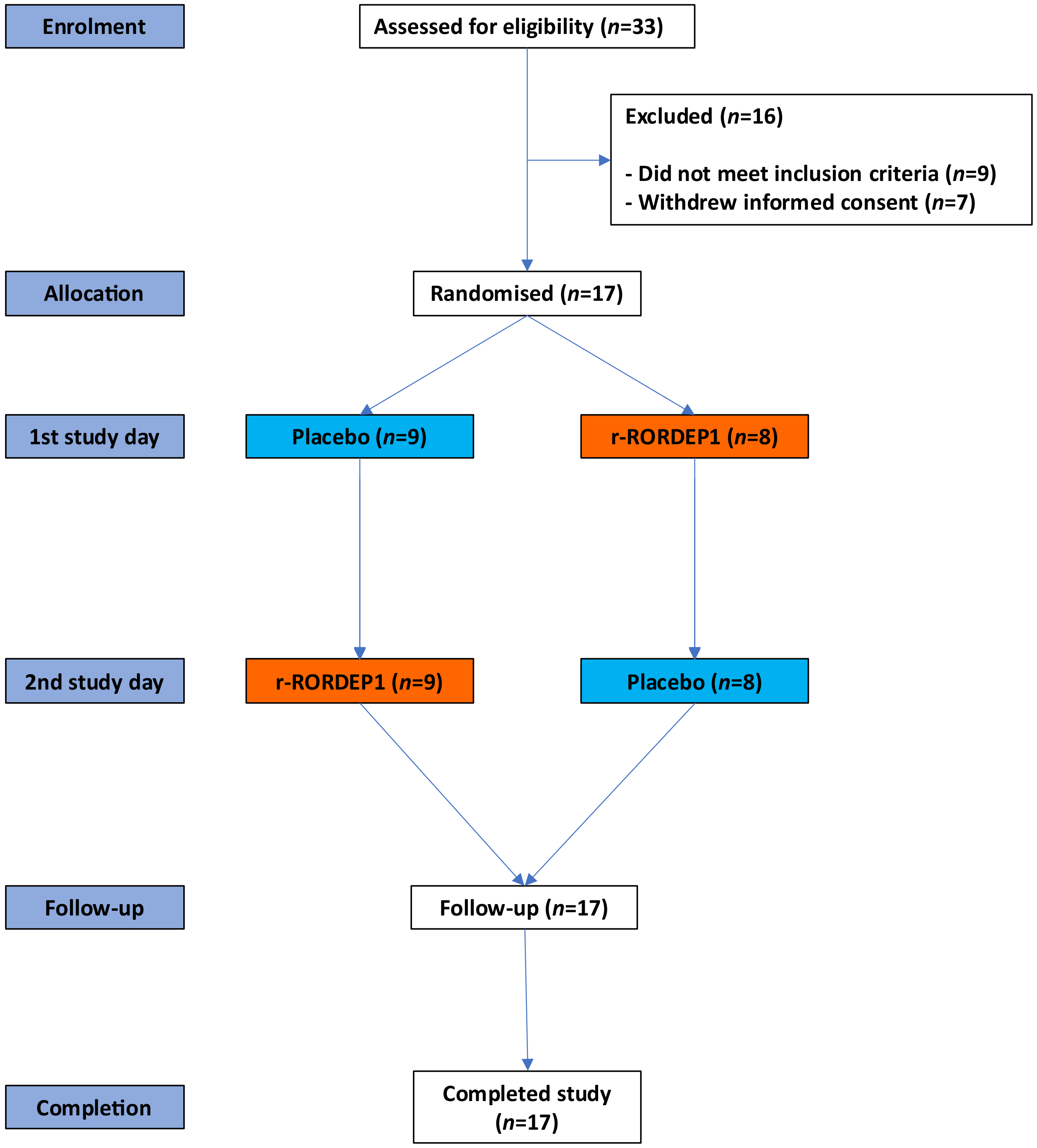
Enrolment
Allocation
1st study day
2nd study day
Follow-up
Completion
Assessed for eligibility (n=33)
Excluded (n=16)
- Did not meet inclusion criteria (n=9)
- Withdrew informed consent (n=7)
Randomised (n=17)
Placebo (n=9)
r-RORDEP1 (n=8)
r-RORDEP1 (n=9)
Placebo (n=8)
Follow-up (n=17)
Completed study (n=17)

Legend to ESM Fig. 1 Flow diagram of participant enrolment and randomisation and cross-over. Study participants were randomised on the first study day through block randomisation. All participants received both interventions with one week between study visits. All randomised participants received both interventions and completed the study. There were no dropouts.

ESM Fig. 2

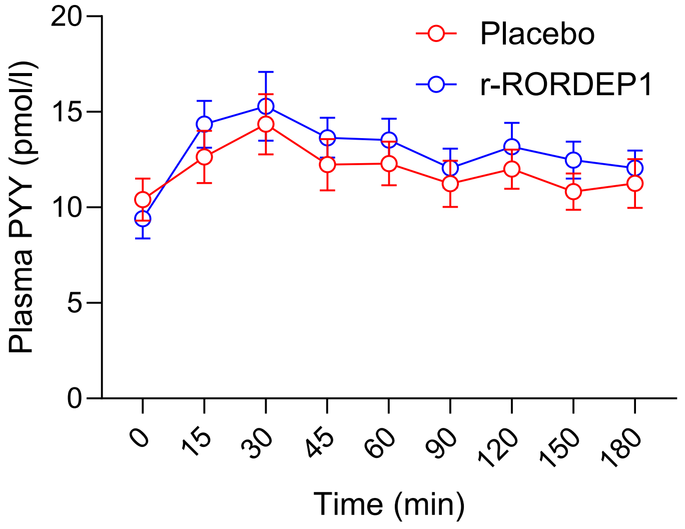

Legend to ESM Fig. 2 Plasma concentrations of PYY during the mixed meal test.

Dots and bars represent mean and standard error of the mean.

PYY, peptide YY.
